# Upper cervical and upper thoracic manipulation versus mobilization and exercise in patients with cervicogenic headache: a multi-center randomized clinical trial

**DOI:** 10.1186/s12891-016-0912-3

**Published:** 2016-02-06

**Authors:** James R. Dunning, Raymond Butts, Firas Mourad, Ian Young, Cesar Fernandez-de-las Peñas, Marshall Hagins, Thomas Stanislawski, Jonathan Donley, Dustin Buck, Todd R. Hooks, Joshua A. Cleland

**Affiliations:** Alabama Physical Therapy & Acupuncture, Montgomery, AL USA; Nova Southeastern University, Ft. Lauderdale, FL USA; AAMT Fellowship in Orthopaedic Manual Physical Therapy, Columbia, SC USA; Research Physical Therapy Specialists, Columbia, SC USA; Universidad Rey Juan Carlos, Alcorcón, Spain; Spine and Sport, Savannah, GA USA; Department of Physical Therapy, Occupational Therapy, Rehabilitation and Physical Medicine, Universidad Rey Juan Carlos, Alcorcón, Spain; Department of Physical Therapy, Long Island University, Brooklyn, NY USA; Back to Health, Brooklyn, NY USA; Cutting Edge Orthopedics, Gilbert, AZ USA; Champion Sports Medicine, Birmingham, AL USA; Department of Physical Therapy, Franklin Pierce University, Manchester, NH USA

**Keywords:** Cervicogenic headache, Spinal manipulation, Mobilization, High velocity low amplitude thrust

## Abstract

**Background:**

Although commonly utilized interventions, no studies have directly compared the effectiveness of cervical and thoracic manipulation to mobilization and exercise in individuals with cervicogenic headache (CH). The purpose of this study was to compare the effects of manipulation to mobilization and exercise in individuals with CH.

**Methods:**

One hundred and ten participants (*n* = 110) with CH were randomized to receive both cervical and thoracic manipulation (*n* = 58) or mobilization and exercise (*n* = 52). The primary outcome was headache intensity as measured by the Numeric Pain Rating Scale (NPRS). Secondary outcomes included headache frequency, headache duration, disability as measured by the Neck Disability Index (NDI), medication intake, and the Global Rating of Change (GRC). The treatment period was 4 weeks with follow-up assessment at 1 week, 4 weeks, and 3 months after initial treatment session. The primary aim was examined with a 2-way mixed-model analysis of variance (ANOVA), with treatment group (manipulation versus mobilization and exercise) as the between subjects variable and time (baseline, 1 week, 4 weeks and 3 months) as the within subjects variable.

**Results:**

The 2X4 ANOVA demonstrated that individuals with CH who received both cervical and thoracic manipulation experienced significantly greater reductions in headache intensity (*p* < 0.001) and disability (*p* < 0.001) than those who received mobilization and exercise at a 3-month follow-up. Individuals in the upper cervical and upper thoracic manipulation group also experienced less frequent headaches and shorter duration of headaches at each follow-up period (*p* < 0.001 for all). Additionally, patient perceived improvement was significantly greater at 1 and 4-week follow-up periods in favor of the manipulation group (*p* < 0.001).

**Conclusions:**

Six to eight sessions of upper cervical and upper thoracic manipulation were shown to be more effective than mobilization and exercise in patients with CH, and the effects were maintained at 3 months.

**Trial registration:**

NCT01580280 April 16, 2012.

## Background

The International Classification of Headache Disorders defines cervicogenic headache (CH) as, “headache caused by a disorder of the cervical spine and its component bony, disc, and/or soft tissue elements, usually but not invariably accompanied by neck pain.” [1] ^(p.760)^ The prevalence of CH has been reported to be between 0.4 and 20 % of the headache population [2, 3], and as high as 53 % in patients with headache after whiplash injury [4]. The dominant features of CH usually include: unilaterality of head pain without side-shift, elicitation of pain with external pressure over the ipsilateral upper neck, limited cervical range of motion, and the triggering of attacks by various awkward or sustained neck movements [4, 5].

Individuals with CH are frequently treated with spinal manipulative therapy including both mobilization and manipulation [6]. Spinal mobilization consists of slow, rhythmical, oscillating techniques whereas manipulation consists of high-velocity low-amplitude thrust techniques. [7] In a recent systematic review, Bronfort and colleagues reported that spinal manipulative therapy (both mobilization and manipulation) were effective in the management of adults with CH [8]. However, they did not report if manipulation resulted in superior outcomes compared to mobilization for the management of this population.

Several studies have investigated the effect of spinal manipulation in the management of CH [9–13]. Haas et al. [10] investigated the effectiveness of cervical manipulation in subjects with CH. Jull et al. [11] demonstrated treatment efficacy for manipulative therapy and/or exercise in the management of CH. However the manipulative therapy group included manipulation and mobilization therefore it cannot be determined if the beneficial effect was a result of the manipulation, mobilization or the combination.

A few studies have examined the benefits of manipulation versus mobilization for the management of mechanical neck pain with or without exercise [14–16]. However, no studies have directly compared the effects of manipulation versus mobilization and exercise in patients with CH. Considering the purported risks of manipulation [17], it is essential to determine if manipulation results in improved outcomes compared to mobilization for the management of patients with CH. Therefore, the purpose of this randomized clinical trial was to compare the effects of manipulation versus mobilization and exercise in patients with CH. We hypothesized that patients receiving manipulation over a 4-week treatment period would experience greater reductions in headache intensity, headache frequency, headache duration, disability, and medication intake at a 3-month follow-up than patients receiving cervical and thoracic mobilization combined with exercise.

## Methods

### Participants

In this multi-center randomized clinical trial, consecutive patients with CH presenting to 1 of 8 outpatient physical therapy clinics from a variety of geographical locations (Arizona, Georgia, New York, Ohio, Pennsylvania, South Carolina) were recruited over a 29-month period (from April 2012 to August 2014). For patients to be eligible, they had to present with a diagnosis of CH according to the revised diagnostic criteria [5] developed by the Cervicogenic Headache International Study Group (CHISG) [5, 18, 19]. CH was classified according to the “major criteria” (not including confirmatory evidence by diagnostic anesthetic blockades) and “head pain characteristics” of the CHISG. Therefore, in order to be included in the study, patients had to exhibit all of the following criteria: (1) unilaterality of the head pain without sideshift, starting in the upper posterior neck or occipital region, eventually spreading to the oculofrontotemporal area on the symptomatic side, (2) pain triggered by neck movement and/or sustained awkward positions, (3) reduced range of motion in the cervical spine [20] (i.e., less than or equal to 32 ° of right or left passive rotation on the Flexion-Rotation Test [21–23], (4) pain elicited by external pressure over at least one of the upper cervical joints (C0-3), and (5) moderate to severe, non-throbbing and non-lancinating pain. In addition, participants had to have a headache frequency of at least 1 per week for a minimum of 3 months, a minimum headache intensity pain score of two points (0–10 on the NPRS scale), a minimum disability score of 20 % or greater (i.e., 10 points or greater on the 0–50 NDI scale), and be between 18 and 65 years of age.

Patients were excluded if they exhibited other primary headaches (i.e., migraine, TTH), suffered from bilateral headaches, or exhibited any red flags (i.e., tumor, fracture, metabolic diseases, rheumatoid arthritis, osteoporosis, resting blood pressure greater than 140/90 mmHg, prolonged history of steroid use, etc.), presented with two or more positive neurologic signs consistent with nerve root compression (muscle weakness involving a major muscle group of the upper extremity, diminished upper extremity deep tendon reflex, or diminished or absent sensation to pinprick in any upper extremity dermatome), presented with a diagnosis of cervical spinal stenosis, exhibited bilateral upper extremity symptoms, had evidence of central nervous system involvement (hyperreflexia, sensory disturbances in the hand, intrinsic muscle wasting of the hands, unsteadiness during walking, nystagmus, loss of visual acuity, impaired sensation of the face, altered taste, the presence of pathological reflexes), had a history of whiplash injury within the previous 6 weeks, had prior surgery to the head or neck, had received treatment for head or neck pain from any practitioner within the previous month, had received physical therapy or chiropractic treatment for head or neck pain within the previous 3 months, or had pending legal action regarding their head or neck pain.

The most recent literature suggests that pre-manipulative cervical artery testing is unable to identify those individuals at risk of vascular complications from cervical manipulation [24, 25], and any symptoms detected during pre-manipulative testing may be unrelated to changes in blood flow in the vertebral artery [26, 27]. Hence, pre-manipulative cervical artery testing was not performed in this study; however, screening questions for cervical artery disease had to be negative [24, 28, 29]. This study was approved by the Institutional Review Board at Long Island University, Brooklyn, NY. The study was registered at www.clinicaltrials.gov with trial identifier NCT01580280. All patients were informed that they would receive either manipulation or mobilization and exercise and then provided informed consent before their enrollment in the study.

### Treating therapists

Twelve physical therapists (mean age 36.6 years, SD 5.62) participated in the delivery of treatment for patients in this study. They had an average of 10.3 (SD 5.66, range 3–20 years) years of clinical experience, and all had completed a 60 h post-graduate certification program that included practical training in manual techniques including the use of cervical and thoracic manipulation. To ensure all examination, outcome assessments, and treatment procedures were standardized, all participating physical therapists were required to study a manual of standard operating procedures and participate in a 4 h training session with the principal investigator.

### Examination procedures

All patients provided demographic information, completed the Neck Pain Medical Screening Questionnaire, and completed a number of self-report measures, followed by a standardized history and physical examination at baseline. Self-report measures included headache intensity as measured by the NPRS (0–10), the NDI (0–50), headache frequency (number of days with headache in the last week), headache duration (total hours of headache in the last week), and medication intake (number of times the patient had taken narcotic or over-the-counter pain medication in the past week).

The standardized physical examination was not limited to, but included measurements of C1-2 (atlanto-axial joint) passive right and left rotation ROM using the Flexion-Rotation Test (FRT). The inter-rater reliability for the FRT has been found to be excellent (ICC: 0.93; 95 % CI: 0.87, 0.96) [30].

### Outcome measures

The primary outcome measure used in this study was the patient’s headache intensity as measured by the NPRS. Patients were asked to indicate the average intensity of headache pain over the past week using an 11-point scale ranging from 0 (“no pain”) to 10 (“worst pain imaginable”) at baseline, 1-week, 1-month, and 3-months following the initial treatment session [31]. The NPRS is a reliable and valid instrument to assess pain intensity [32–34]. Although no data exists in patients with CH, the MCID for the NPRS has been shown to be 1.3 in patients with mechanical neck pain [32] and 1.74 in patients with a variety of chronic pain conditions [34]. Therefore, we chose to only include patients with an NPRS score of 2 points (20 %) or greater.

Secondary outcome measures included the NDI, the Global Rating of Change (GRC), headache frequency, headache duration, and medication intake. The NDI is the most widely used instrument for assessing self-rated disability in patients with neck pain [35–37]. The NDI is a self-report questionnaire with 10-items rated from 0 (no disability) to five (complete disability) [38]. The numeric responses for each item are summed for a total score ranging between 0 and 50; however, some evaluators have chosen to multiply the raw score by two, and then report the NDI on a 0–100 % scale [36, 39]. Higher scores represent increased levels of disability. The NDI has been found to possess excellent test-retest reliability, strong construct validity, strong internal consistency and good responsiveness in assessing disability in patients with mechanical neck pain [36], cervical radiculopathy [33, 40], whiplash associated disorder [38, 41, 42], and mixed non-specific neck pain [43, 44]. Although no studies have examined the psychometric properties of the NDI in patients with CH, we chose to only include patients with an NDI score of ten points (20 %) or greater, because this cut-off score captures the MCID for the NDI, which has been reported to approximate four, eight, and nine points (0–50) in patients with mixed non-specific neck pain [44], mechanical neck pain [45], and cervical radiculopathy [33], respectively. Headache frequency was measured as the number of days with headache in the last week, ranging from 0 to 7 days. Headache duration was measured as the total hours of headache in the last week, with six possible ranges: (1) 0–5 h, (2) 6–10 h, (3) 11–15 h, (4) 16–20 h, (5) 21–25 h, or (6) 26 or more hours. Medication intake was measured as the number of times the patient had taken prescription or over-the-counter analgesic or anti-inflammatory medication in the past week for their headaches, with five options: (1) not at all, (2) once a week, (3) once every couple of days, (4) once or twice a day, or (5) three or more times a day.

Patients returned for 1-week, 4-weeks, and 3-months follow-ups where the aforementioned outcome measures were again collected. In addition, at the 1-week, 4-weeks and 3-months follow-ups, patients completed a 15-point GRC question based on a scale described by Jaeschke et al. [46] to rate their own perception of improved function. The scale ranges from -7 (a very great deal worse) to zero (about the same) to +7 (a very great deal better). Intermittent descriptors of worsening or improving are assigned values from -1 to -6 and +1 to +6, respectively. The MCID for the GRC has not been specifically reported but scores of +4 and +5 have typically been indicative of moderate changes in patient status [46]. However, it should be noted that recently Schmitt and Abbott reported that the GRC might not correlate with changes in function in a population with hip and ankle injuries [47]. All outcome measures were collected by an assessor blind to group assignment.

On the initial visit patients completed all outcome measures then received the first treatment session. Patients completed 6–8 treatment sessions of either manipulation or mobilization combined with exercise over 4 weeks. Additionally, subjects were asked if they had experienced any “major” adverse events [48, 49] (stroke or permanent neurological deficits) at each follow-up period.

### Randomization

Following the baseline examination, patients were randomly assigned to receive either manipulation or mobilization and exercise. Concealed allocation was performed by using a computer-generated randomized table of numbers created by an individual not involved with recruiting patients prior to the beginning of the study. Individual, sequentially numbered index cards with the random assignment were prepared for each of 8 data collection sites. The index cards were folded and placed in sealed opaque envelopes. Blinded to the baseline examination, the treating therapist opened the envelope and proceeded with treatment according to the group assignment. Patients were instructed not to discuss the particular treatment procedure received with the examining therapist. The examining therapist remained blind to the patient’s treatment group assignment at all times; however, based on the nature of the interventions it was not possible to blind patients or treating therapists.

### Manipulation group

**Fig. 1:**
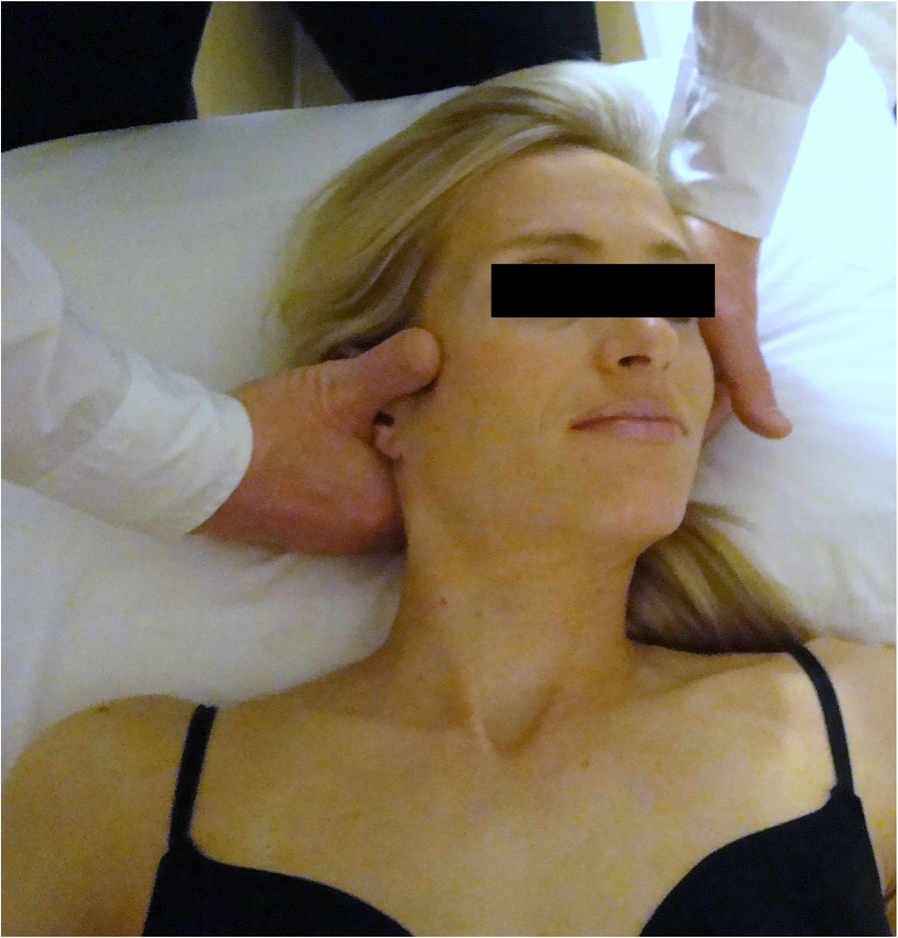
High-velocity low-amplitude thrust manipulation directed to the right C1-2 articulation. The subject provided consent for her image to be used

**Fig. 2:**
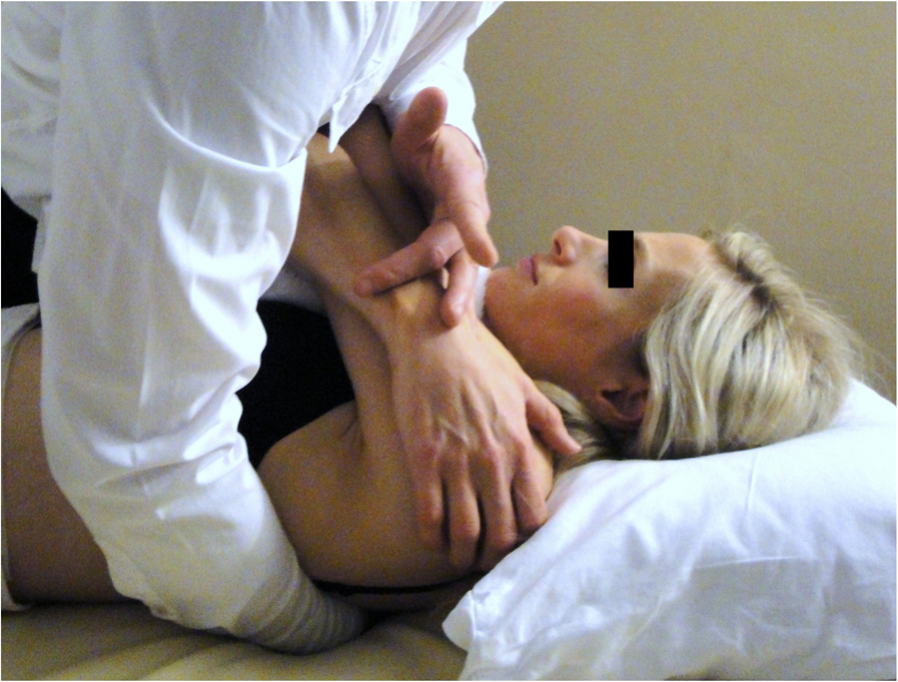
High-velocity low-amplitude thrust manipulation directed bilaterally to the upper thoracic (T1-2) spine. The subject provided consent for her image to be used

Manipulations targeting the right and left C1-2 articulations and bilateral T1-2 articulations were performed on at least one of the 6–8 treatment sessions (Figs. 1 and 2). On other treatment sessions, therapists either repeated the C1-2 and/or T1-2 manipulations or targeted other spinal articulations (i.e., C0-1, C2-3, C3-7, T2-9, ribs 1–9) using manipulation. The selection of the spinal segments to target was left to the discretion of the treating therapist and it was based on the combination of patient reports and manual examination. For both the upper cervical and upper thoracic manipulations, if no popping or cracking sound was heard on the first attempt, the therapist repositioned the patient and performed a second manipulation. A maximum of 2 attempts were performed on each patient similar to other studies [14, 50–53]. The clinicians were instructed that the manipulations are likely to be accompanied by multiple audible popping sounds [54–58]. Patients were encouraged to maintain usual activity within the limits of pain; however, mobilization and the prescription of exercises, or any use of other modalities, were not provided to this group.

The manipulation targeting C1-2 was performed with the patient in supine. For this technique, the patient’s left posterior arch of the atlas was contacted with the lateral aspect of the proximal phalanx of the therapist’s left second finger using a “cradle hold”. To localize the forces to the left C1-2 articulation, the patient was positioned using extension, a posterior-anterior (PA) shift, ipsilateral side-bend and contralateral side-shift. While maintaining this position, the therapist performed a single high-velocity, low-amplitude thrust manipulation to the left atlanto-axial joint using right rotation in an arc toward the underside eye and translation toward the table (Fig. 1). This was repeated using the same procedure but directed to the right C1-2 articulation.

The manipulation targeting T1-2 was performed with the patient in supine. For this technique, the patient held her/his arms and forearms across the chest with the elbows aligned in a superoinferior direction. The therapist contacted the transverse processes of the lower vertebrae of the target motion segment with the thenar eminence and middle phalanx of the third digit. The upper lever was localized to the target motion segment by adding rotation away and side-bend towards the therapist while the underside hand used pronation and radial deviation to achieve rotation toward and side-bend away moments, respectively. The space inferior to the xiphoid process and costochondral margin of the therapist was used as the contact point against the patient’s elbows to deliver a manipulation in an anterior to posterior direction targeting T1-2 bilaterally (Fig. 2).

### Mobilization and exercise group

Mobilizations targeting the right and left C1-2 articulations and bilateral T1-2 articulations were performed on at least one of the 6–8 treatment sessions. On other treatment sessions, therapists either repeated the C1-2 and/or T1-2 mobilizations or targeted other spinal articulations (i.e., C0-1, C2/3, C3-7, T2-9, ribs 1–9) using mobilization. The selection of the spinal segments to target was left to the discretion of the treating therapist and it was based on the combination of patient reports and manual examination. However, in order to avoid a “contact” or “attention effect” when compared with the manipulation group, therapists were instructed to mobilize one cervical segment (i.e., right and left) and one thoracic segment or rib articulation on each treatment session.

The mobilization targeting the C1-2 articulation was performed in prone. For this technique, the therapist performed one 30 s bout of left-sided unilateral grade IV PA mobilizations to the C1-2 motion segment as described by Maitland [7]. This same procedure was repeated for one 30 s bout to the right atlanto-axial joint. In addition, and on at least one session, mobilization directed to the upper thoracic (T1-2) spine with the patient prone was performed. For this technique, the therapist performed one 30 s bout of central grade IV PA mobilizations to the T1-2 motion segment as described by Maitland [7]. Therefore, we used 180 (i.e., three 30 s bouts at approximately 2 Hz) end-range oscillations in total on each subject for the mobilization treatment. Notably, there is no high quality evidence to date to suggest that longer durations of mobilization result in greater pain reduction than shorter durations or dosages of mobilization [59, 60].

Cranio-cervical flexion exercises [11, 61–63] were performed with the patient in supine, with the knees bent and the position of the head standardized by placing the craniocervical and cervical spines in a mid-position, such that a line between the subject’s forehead and chin was horizontal, and a horizontal line from the tragus of the ear bisected the neck longitudinally. An air-filled pressure biofeedback unit (Chattanooga Group, Inc., Hixson, TN) was placed suboccipitally behind the patient’s neck and preinflated to a baseline of 20 mmHg [63]. For the staged exercises, patients were required to perform the craniocervical flexion action (“a nod of the head, similar to indicating yes”) [63] and attempt to visually target pressures of 22, 24, 26, 28, and 30 mmHg from a resting baseline of 20 mmHg and to hold the position steady for 10 s [61, 62]. The action of nodding was performed in a gentle and slow manner. A 10 s rest was allowed between trials. If the pressure deviated below the target pressure, the pressure was not held steady, substitution with the superficial flexors (sternocleidomastoid or anterior scalene) occurred, or neck retraction was noticed before the completion of the 10 s isometric hold, it was regarded as a failure [63]. The last successful target pressure was used to determine each patient’s exercise level wherein 3 sets of 10 repetitions with a 10 s isometric hold were performed. In addition to mobilizations and cranio-cervical flexion exercises, patients were required to perform 10 min of progressive resistance exercises (i.e., using Therabands® or free weights) to the muscles of the shoulder girdle during each treatment session, within their own tolerance, and specifically focusing on the lower trapezius and serratus anterior [11].

### Sample size

The sample size and power calculations were performed using online software from the MGH Biostatistics Center (Boston, MA). The calculations were based on detecting a 2-point (or 20 %) difference in the NPRS (headache intensity) at the 3 months follow-up, assuming a standard deviation of three points, a 2-tailed test, and an alpha level equal to 0.05. This generated a sample size of 49 patients per group. Allowing for a conservative dropout rate of 10 %, we planned to recruit at least 108 patients into the study. This sample size yielded greater than 90 % power to detect a statistically significant change in the NPRS scores.

### Data analysis

Descriptive statistics, including frequency counts for categorical variables and measures of central tendency and dispersion for continuous variables were calculated to summarize the data. The effects of treatment on headache intensity and disability were each examined with a 2-by-4 mixed-model analysis of variance (ANOVA), with treatment group (manipulation versus mobilization and exercise) as the between-subjects variable and time (baseline, 1 week, 4 weeks, and 3 months follow-up) as the within-subjects variable. Separate ANOVAs were performed with the NPRS (headache intensity) and NDI (disability) as the dependent variable. For each ANOVA, the hypothesis of interest was the 2-way interaction (group by time).

An independent *t*-test was used to determine the between group differences for the percentage change from baseline to 3-month follow-up in both headache intensity and disability. Separate Mann–Whitney *U* tests were performed with the headache frequency, GRC, headache duration and medication intake as the dependent variable. We performed Little’s Missing Completely at Random (MCAR) test [64] to determine if missing data points associated with dropouts were missing at random or missing for systematic reasons. Intention-to-treat analysis was performed by using Expectation-Maximization whereby missing data are computed using regression equations. Planned pairwise comparisons were performed examining the difference between baseline and follow-up periods between-groups using the Bonferroni correction at an alpha level of .05.

We dichotomized patients as responders at the 3-month follow-up using a cut score of 2 points improvement for headache intensity as measured by the NPRS. Numbers needed to treat (NNT) and 95 % confidence intervals (CI) were also calculated at the 3 months follow-up period using each of these definitions for a successful outcome. Data analysis was performed using SPSS 21.0.

## Results

**Fig. 3:**
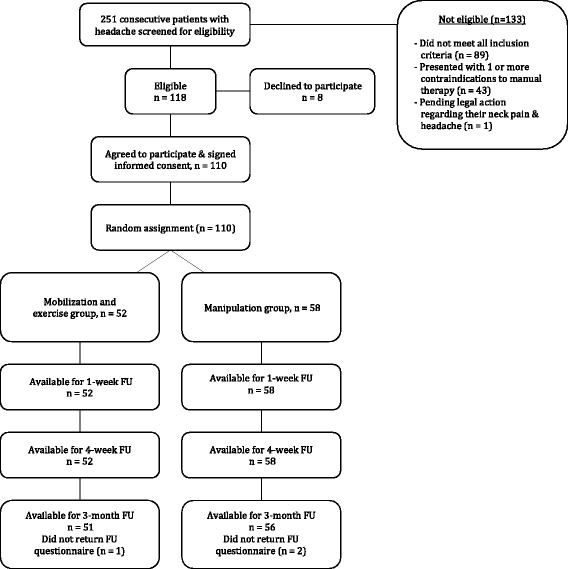
Flow diagram of patient recruitment and retention

Two hundred and fifty-one patients with a primary complaint of headaches were screened for possible eligibility. The reasons for ineligibility can be found in Fig. 3, the flow diagram of patient recruitment and retention. Of the 251 patients screened, 110 patients, with a mean age of 35.16 years (SD 11.48) and a mean duration of symptoms of 4.56 years (SD 6.27), satisfied the eligibility criteria, agreed to participate, and were randomized into manipulation (*n* = 58) and mobilization and exercise (*n* = 52) groups. Baseline variables for each group can be found in Table 1. Twelve therapists from 8 outpatient physical therapy clinics each treated 25, 23, 20, 14, 13, 7, 6 or 2 patients, respectively; furthermore, each of the 12 therapists treated approximately an equal proportion of patients in each group. There was no significant difference (*p* = 0.227) between the mean number of completed treatment sessions for the manipulation group (7.17, SD 0.96) and the mobilization and exercise group (6.90, SD 1.35). In addition, the mean number of treatment sessions that targeted the C1-2 articulation was 6.41 (SD 1.63) for the manipulation group and 6.52 (SD 2.01) for the mobilization and exercise group, and this was not significantly different (*p* = 0.762). One hundred seven of the 110 patients completed all outcome measures through 3 months (97 % follow-up). Little’s Missing Completely at Random (MCAR) test was not statistically significant (*p =* 0.281); therefore, we used the Expectation-Maximization imputation technique to replace missing values with predicted values for the missing 3-month outcomes.

**Table 1:** Baseline variables: demographics and outcome measures

Baseline Variable	Manipulation Group (*n* = 58)	Mobilization & Exercise Group (*n* = 52)
Age (years): Mean (SD)	34.1 (12.6)	36.4 (10.0)
Gender (female): number (%)	41 (71 %)	33 (64 %)
Duration of symptoms (days): Mean (SD)	1693.7 (2357.7)	1633.8 (2229.9)
BMI (kg/m^2^): Mean (SD)	24.2 (3.8)	24.0 (3.3)
Headache intensity (NPRS 0–10): Mean (SD)	6.4 (1.6)	6.0 (2.1)
Disability (NDI 0–50): Mean (SD)	18.1 (7.9)	19.2 (7.8)
Headache frequency (0–7 days): Median	4	4
Headache duration: Median	3	3
Medication intake: Median	3	3

*NPRS* Numeric Pain Rating Scale, 0–10, lower scores indicate less pain; *NDI* Neck Disability Index, *0*–*50*, lower scores indicate greater function; Headache frequency = number of headache days in the last week, 0–7, higher scores indicate worsening; Headache duration = total headache hours in the last week, 1 = 0–5 h, 2 = 6–10 h, 3 = 11–15 h, 4 = 16–20 h, 5 = 21–25 h, 6 = 26 or more hours, higher scores indicate worsening; Medication intake = frequency of pain medication use in the past week, 1 = not at all, 2 = once a week, 3 = once every couple of days, 4 = once or twice a day, 5 = three or more times a day

The overall group by time interaction for the primary outcome of headache intensity was statistically significant for the NPRS (F_(3,106)_ = 11.196; *p* < 0.001; partial eta squared = 0.24). Between-group differences revealed that the manipulation group experienced statistically significant greater improvement in the NPRS at both the 1-week (2.1, 95 % CI: 1.2, 2.9), 4-week (2.3, 95 % CI: 1.5, 3.1) and 3-month (2.1, 95 % CI: 1.2, 3.0) follow-up periods (Table 2). In addition, an independent samples *t-*test revealed the between-group difference in percentage change in headache intensity (36.58 %, 95 % CI: 22.52, 50.64) from baseline to 3-month follow-up was statistically significant (t_(108)_ = 5.156; *p* < 0.001) in favor of manipulation. See Table 3 for the percentage of subjects gaining 50, 75, and 100 % reduction in headache intensity at 3 months.

**Table 2:** Changes in headache intensity (NPRS) and disability (NDI) with 95 % confidence intervals for both groups and between-group differences

Variable	Manipulation	Mobilization and Exercise	Between-Group Differences
Headache Intensity (NPRS 0–10)			
Baseline: Mean (SD)	6.4 (1.6)	6.0 (2.1)	
1-Week: Mean (SD)	3.1 (1.9)	4.9 (1.8)	
Change Score: Baseline to 1-Week	3.2 (2.6, 3.8)	1.2 (0.6, 1.7)	2.1 (1.2, 2.9); *P* < 0.001
4-Week: Mean (SD)	1.8 (1.6)	3.8 (2.0)	
Change Score: Baseline to 4-Week	4.5 (4.0, 5.1)	2.2 (1.7, 2.8)	2.3 (1.5, 3.1); *P* < 0.001
3-Month: Mean (SD)	2.0 (1.8)	3.8 (1.9)	
Change Score: Baseline to 3-Month	4.3 (3.7, 4.9)	2.2 (1.6, 2.9)	2.1 (1.2, 3.0); *P* < 0.001
Disability (NDI 0–50)			
Baseline: Mean (SD)	18.1 (7.9)	19.2 (7.8)	
1-Week: Mean (SD)	11.9 (8.5)	16.1 (7.5)	
Change Score: Baseline to 1-Week	6.2 (4.8, 7.6)	3.1 (2.0, 4.1)	3.1 (1.4, 4.9); *P* < 0.001
4-Week: Mean (SD)	6.5 (5.4)	13.0 (7.5)	
Change Score: Baseline to 4-Week	11.6 (9.7, 13.4)	6.1 (4.9, 7.4)	5.4 (3.2, 7.7); *P* < 0.001
3-Month: Mean (SD)	6.3 (5.9)	13.5 (7.8)	
Change Score: Baseline to 3-Month	11.7 (9.7, 13.8)	5.7 (4.2, 7.2)	6.0 (3.5, 8.6); *P* < 0.001

*NPRS* Numeric Pain Rating Scale, 0–10, lower scores indicate less pain; *NDI* Neck Disability Index, 0–50, lower scores indicate greater function

**Table 3:** Percentage of subjects gaining 50, 75 and 100 % reduction in headache intensity (NPRS) and disability (NDI) as well as the numbers needed to treat at 3 months

Variable	Manipulation (*n* = 58)	Mobilization & Exercise (*n* = 52)
Headache Intensity (NPRS 0–10)		
50 % Reduction	74.1 %	38.5 %
75 % Reduction	48.3 %	13.5 %
100 % Reduction	29.3 %	3.8 %
Number of individuals achieving at least a 2 point improvement in pain	53	33
Numbers Needed to Treat	4.0 (95 % CI: 2.3, 7.7)	
Disability (NDI 0–50)		
50 % Reduction	74.1 %	23.1 %
75 % Reduction	43.1 %	9.6 %
100 % Reduction	19.0 %	1.9 %

For secondary outcomes a significant group by time interaction existed for the NDI (F_(3,106)_ = 8.57; *p* < 0.001; partial eta squared = 0.20). At each follow-up period the manipulation group had superior outcomes in disability reduction as compared to the mobilization and exercise group. An independent samples *t-* test revealed the between-group mean percentage change in disability (35.56 %, 95 % CI: 24.95, 46.17) from baseline to 3 months follow-up was statistically significant (t_(108)_ = 6.646, *p* < 0.001); indicating the manipulation group experienced a significantly greater percentage in disability reduction (Table 3).

Mann–Whitney *U* tests revealed that patients in the upper cervical and upper thoracic manipulation group experienced less frequent headaches at 1 week (*p* < 0.001; median 2.0 versus 3.0), 4 weeks (*p* < 0.001; median 1.0 versus 3.0) and 3 months (*p* < 0.001; median 1.0 versus 2.5) than patients in the mobilization and exercise group. Headache duration was significantly lower at 1 week (*p* = 0.005; median 2.0 versus 3.0, 4 weeks (*p* < 0.001; median 1.0 versus 2.0) and 3 months (*p* < 0.001; median 1.0 versus 2.0) in the manipulation group. Additionally, patient perceived improvement as measured by the GRC was significantly greater at 1 week (*p* < 0.001, 4.0 versus 1.0), 4 weeks (*p* < 0.001, 6.0 versus 3.0) and 3 months (*p* < 0.001, 6.0 versus 3.0) than patients in the mobilization and exercise group. At 3 months, patients receiving upper cervical and upper thoracic manipulation experienced significantly (*p* < 0.001) greater reductions in medication intake as compared to the mobilization and exercise group. Based on the cutoff score of 2 points on the NPRS, the NNT was 4.0 (95 % CI: 2.3, 7.7) in favor of the manipulation group at 3-month follow-up.

We did not collect any data on the occurrence of “minor” adverse events [48, 49] (transient neurological symptoms, increased stiffness, radiating pain, fatigue or other); however, no “major” adverse events [48, 49] (stroke or permanent neurological deficits) were reported for either group.

## Discussion

### Statement of principal findings

To our knowledge, this study is the first randomized clinical trial to directly compare the effectiveness of both cervical and thoracic manipulation to mobilization and exercise in patients with CH. The results suggest 6–8 sessions of manipulation over 4 weeks, directed mainly to both the upper cervical (C1-2) and upper thoracic (T1-2) spines, resulted in greater improvements in headache intensity, disability, headache frequency, headache duration, and medication intake than mobilization combined with exercises. The point estimates for between-group changes in headache intensity (2.1 points) and disability (6.0 points or 12.0 %) exceeded the reported MCIDs for both measures. Although the MCID for the NDI in patients with CH has not yet been investigated, it should however be noted that the lower bound estimate of the 95 % CI for disability (3.5 points) was slightly below (or approximated in two cases) the MCID that has been found to be 3.5 [65], 5 [66], and 7.5 [45] points in patients with mechanical neck pain, 8.5 [33] points in patients with cervical radiculopathy, and 3.5 [44] points in patients with mixed, non-specific neck pain. However, it should be recognized that both groups made clinical improvement. In addition, the NNT suggests for every four patients treated with manipulation, rather than mobilization, one additional patient achieves clinically important pain reduction at 3 months follow-up.

### Strengths and weaknesses of the study

The inclusion of 12 treating physical therapists from 8 private clinics in 6 different geographical states enhances the overall generalizability of our findings. Although significant differences were recognized up to 3 months, it is not known if these benefits would have been sustained at long-term. In addition, we used high-velocity, low-amplitude manipulation techniques that employed bidirectional thrusts into rotation and translation simultaneously and Maitland based grade IV PA mobilization techniques; thus, we cannot be certain that these results are generalizable to other kinds of manual therapy techniques. Some might argue that the comparison group might have not received adequate intervention. We sought to balance internal and external validity so standardized treatment for both groups and provided a very explicit description of the techniques used which will also allow for replication. Furthermore, we did not measure minor adverse events and only asked about two potential major adverse events. Another limitation is that we included multiple secondary outcomes. Therapist preferences as to which technique they thought would be superior was not collected and potentially could impact the results.

### Strengths and weaknesses in relation to other studies: important differences in results

Jull et al. [11] demonstrated treatment efficacy for manipulative therapy and exercise in the management of CH; however, this treatment package included both mobilization and manipulation. The current study may provide evidence that the management of patients with CH should include some form of manipulation despite the fact it is often suggested that cervical manipulation should be avoided because of the risk of serious adverse events [67, 68]. Furthermore, it has been shown that individuals receiving spinal manipulation for neck pain and headaches are no more likely to experience a vertebrobasilar stroke than if they received treatment by their medical physician [69]. Additionally, after reviewing 134 case reports, Puentedura et al. concluded that with appropriate selection of patients by careful screening of red flags and contraindications, the majority of adverse events associated with cervical manipulation could have been prevented [70].

### Meaning of the study: possible explanations and implications for clinicians and policymakers

Based on the results of the current study clinicians should consider incorporating spinal manipulation for individuals with CH. A recent systematic review found both mobilization and manipulation to be effective for the management of patients with CH but was unable to determine which technique was superior [8]. Additionally, clinical guidelines reported that manipulation, mobilization and exercise were all effective for the management of patients with CH; however, the guideline made no suggestions regarding the superiority of either technique. [71] The current results may assist authors of future systematic reviews and clinical guidelines in providing more specific recommendations about the use of spinal manipulation in this population.

### Unanswered questions and future research

The underlying mechanisms as to why manipulation may have resulted in greater improvements remains to be elucidated. It has been suggested that high-velocity displacement of vertebrae with impulse durations of less than 200 ms may alter afferent discharge rates [72] by stimulating mechanoreceptors and proprioceptors, thereby changing alpha motorneuron excitability levels and subsequent muscle activity [72–74]. Manipulation might also stimulate receptors in the deep paraspinal musculature, and mobilization might be more likely to facilitate receptors in the superficial muscles [75]. Biomechanical [76, 77], spinal or segmental [78, 79] and central descending inhibitory pain pathway [80–83] models are plausible explanations for the hypoalgesic effects observed following manipulation. Recently, the biomechanical effects of manipulation have been under scientific scrutiny [84], and it is plausible that the clinical benefits found in our study are associated with a neurophysiological response involving temporal sensory summation at the dorsal horn of the spinal cord [78]; however, this proposed model is currently supported only on findings from transient, experimentally induced pain in healthy subjects [85, 86], not patients with CH. Future studies should examine different manual therapy techniques with varying dosages and include a 1-year follow-up. Furthermore, future studies examining the neurophysiological effects of both manipulation and mobilization will be important for determining why there may or may not be a difference in clinical effects between these two treatments.

## Conclusion

The results of the current study demonstrated that patients with CH who received cervical and thoracic manipulation experienced significantly greater reductions in headache intensity, disability, headache frequency, headache duration, and medication intake as compared to the group that received mobilization and exercise; furthermore, the effects were maintained at 3 months follow-up. Future studies should examine the effectiveness of different types and dosages of manipulation and include a long-term follow-up.
